# Near-Field Nano-Focusing and Nano-Imaging of Dielectric Microparticle Lenses

**DOI:** 10.3390/nano14231974

**Published:** 2024-12-09

**Authors:** Jinzhong Ling, Yucheng Wang, Jinkun Guo, Xin Liu, Xiaorui Wang

**Affiliations:** School of Optoelectronic Engineering, Xidian University, Xi’an 710071, China; cywang@xidian.edu.cn (Y.W.); xinliu@mail.xidian.edu.cn (X.L.)

**Keywords:** microscopic imaging, super-resolution, microparticle, FDTD algorithm

## Abstract

Compared with traditional far-field objective lenses, microparticle lenses have a distinct advantage of nonobservance of the diffraction limit, which has attracted extensive attention for its application in subwavelength photolithography and super-resolution imaging. In this article, a complete simulation model for a microparticle lens assisted microscopic imaging system was built to analyze the imaging characteristics of any shape of microparticle lens. With this model, we simulated the resolution of a conventional objective lens, a microsphere lens and a hollow microsphere lens, which verified the correctness of our simulation model and demonstrated the super-resolution imaging ability of microsphere lenses. Secondly, the focusing and imaging characteristics of four typical microparticle lenses are illustrated, and how the focal spot affects imaging resolution and imaging quality is analyzed. Upon this conclusion, we reformed and upgraded the microsphere lens with several parameters for smaller focal spots and higher imaging resolution. Finally, three types of microparticle lenses were designed through the optimized parameters and their focusing and imaging characteristics were demonstrated with a minimum FWHM of 140 nm at the focal plane and a highest imaging resolution around 70 nm (~λ/6). Our work opens up a new perspective of super-resolution imaging with near-field microparticle lens.

## 1. Introduction

Research on the focusing performance of microparticle lenses can be traced back to 1908, when Gustav Mie conducted a large number of calculations on the scattering of electromagnetic waves by spherical microparticles in a uniform medium, analyzing the intensity distribution in the near and far fields. However, due to the limited computational power at that time, only a few simple shapes of microparticles’ scattering had been solved with analytical solutions. Until recent decades, with the emergence of numerical calculation methods and the improvement of computational power, scientists have calculated the near-field focusing characteristics of various shapes of microparticle lenses, such as ellipsoids [1,2], cuboids [3,4,5], pyramids [6], triangular prisms [7], Janus particles [8], hollow particles [9,10], and other non-spherical particles [11], which could be applied in many fields, such as nanolithography [12,13], Raman enhancement [14,15], and super-resolution imaging [16,17,18,19,20,21,22,23,24,25,26,27,28].

Since the first discovery of imaging resolution enhancement by microsphere lens attached on the surface of imaging targets by Zengbo Wang et al. in 2011 [16], more and more research has been conducted on its mechanism [17,18,19,20], upgrading [21,22,23,24,25,26], and applications [27,28]. In order to explain this newly discovered phenomenon, researchers analyzed it from the perspectives of Mie scattering [17], evanescent wave [18], whispering gallery mode (WGM) [19], nanojet [20], and virtual image [16], which laid a theoretical foundation for the optimal design of microparticle lens. Although researchers have different understandings of the super-resolution imaging mechanism, it does not hinder the development and popularization of this technology. Firstly, to control the microparticle lens more accurately, researchers have proposed a variety of schemes, such as sucking the microparticle lens with a micropipette for scanning imaging [21], dragging the microparticle with a microfiber [22], swimming the microparticle with untethered chemically power [23], or pushing the microparticle lens with the cantilever of a AFM probe [24]. Secondly, the combination of a microparticle lens with a confocal microscope [25] and dark field microscope [26,27] can improve their imaging resolution, respectively. Meanwhile, the application of microsphere assisted imaging in biological imaging [28] and semiconductor detection [29] shows its powerful super-resolution imaging ability and application potential.

Although the microparticle lens has achieved great super-resolution imaging results, its performance still has great room for improvement. For example, the decoration of micro/nano structure at the microparticle surface can further reduce its focal spot size [30,31]. Currently, most work on optimization and upgrading of microparticle lenses is based on their focusing characteristics, such as the maximum intensity, focal length, and the full width at half maximum (FWHM) of focal spot, but the direct imaging simulation models are still scarce. Therefore, a complete imaging simulation model is still necessary and valuable, which could demonstrate the imaging results more accurately and intuitively and could be used to investigate the relationship between the nano-focusing and nano-imaging performance of microparticle lenses. In this article, a complete imaging simulation model of a microparticle lens assisted microscopic system was constructed for imaging demonstration and microparticle lens optimization. Firstly, we verified the correctness of this simulation model with standard imaging targets and compared the imaging resolution of an objective lens with and without the assistance of a microparticle lens. Secondly, through four specific cases, we analyzed the influence of focal spot characteristics on imaging quality and established the internal relationship between the focusing and imaging. Finally, three types of microparticle lenses were optimized for a smaller focal spot and higher imaging resolution, and their sub-wavelength focusing ability as well as their super-resolution imaging ability have been demonstrated numerically. Particularly, a super-narrow focal spot with the FWHM of 140 nm and a highest resolution around 70 nm have been realized through a hollow microsphere lens. Our research reveals the super-resolution imaging mechanism of the microsphere/microparticle lens and proposes a feasible method for imaging resolution further improvement with a microparticle lens.

## 2. Simulation on Microparticle Lens Assisted Microscopic Imaging

**Figure 1:**
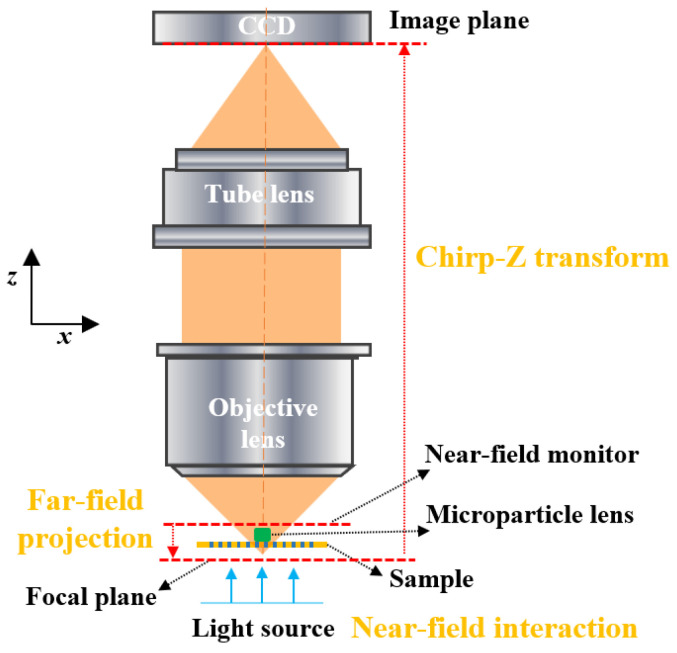
Simulation model for microparticle assisted nanoscopy.

A complete simulation model of a microparticle lens assisted imaging system should include three parts: the focusing of incident light by the microparticle lens, the interaction of the focused light with nano-structures on the sample surface, and the collection of the scattered light by the objective lens, which is a complicated multiscale physical process involving both geometrical and near-field optics given that the dimension of a microparticle is about 10 times that of the wavelength while the dimension of nano-structures on imaging target is below the scale of the wavelength. As illustrated in Figure 1, the simulation process can be completed in three steps: simulating the near-field interaction between the illumination light, the microparticle lens, and the nano-structures at sample surface with the FDTD algorithm; calculating the far-field projection from the near-field monitor to the focal plane of objective lens with angular spectrum theory; simulating the light field propagation from the focal plane to the image plane passing through the objective lens and tube lens by Chirp-Z transform.

**Figure 2:**
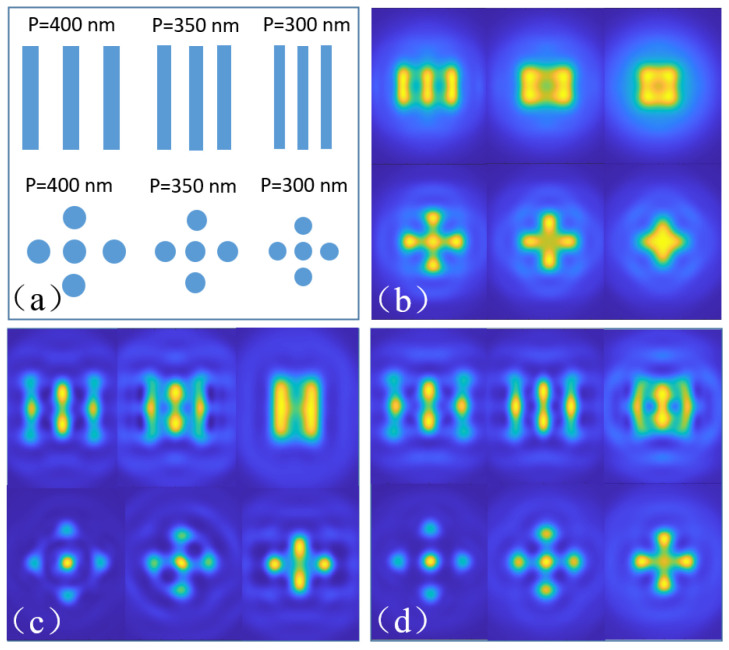
Imaging resolution comparison between convention objective lens and microparticle lens. (**a**) Imaging target with different periods; (**b**) imaging results obtained by an objective lens with NA = 0.9; (**c**) imaged by a microsphere assisted objective lens; (**d**) image obtained by a hollow microsphere.

To verify the correctness of this imaging simulation model, two types of samples (three-bar and five-dot structures) with different periods of 400 nm, 350 nm, and 300 nm are implemented for resolution calibration, as illustrated in Figure 2a. When illuminated by an *x* polarized plane wave with wavelength of 550 nm, and captured by an objective lens with numerical aperture (NA) of 0.9, the structures with a period of 400 nm are clearly imaged, as shown in Figure 2b, while those with a 350 nm and 300 nm period are indistinguishable, which is consistent with the diffraction limit (ε = 0.61λ/NA = 373 nm). In comparison, if assisted with a microsphere lens (D = 4 μm, n = 1.5) at the sample surface, the objective lens’ resolution could be improved significantly, as illustrated in Figure 2c where the structures with periods bigger than 350 nm are distinguishable, and only the ones with a period of 300 are imaged blurry and irresolvable. Additionally, when replaced by a hollow microsphere with same size and same refractive index (RI) but a vacuum core (inner diameter d = 1 μm, n = 1.0), the images become clearer and even the minimum feature size in the structures can be barely distinguished, as shown in Figure 2d. The above results show the correctness of our imaging simulation model and indicate the resolution improvement by a microsphere lens and a hollow microsphere lens.

## 3. Inner Link Between the Nano-Focusing and Nano-Imaging of Microparticle Lens

**Figure 3:**
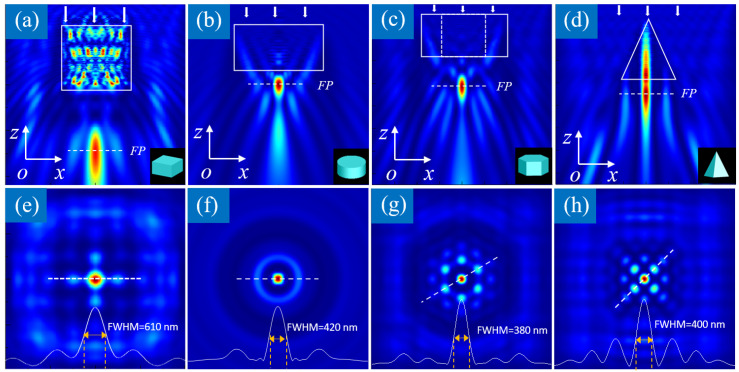
Nano-focusing of different shapes’ microparticle lenses with different FWHMs and sidelobes. (**a**) A micro-cube with sides of 3 μm and an RI of 1.8; (**b**) A micro-cylinder with a radius of 2 μm, a height of 2 μm and an RI of 1.4; (**c**) A hexagonal prism with sides of 2 μm, a height of 2 μm and an RI of 1.3; (**d**) A rectangular pyramid with sides of 3 μm, a height of 3 μm and an RI of 1.4. (**e**) Focal spot of the micro-cube with a FWHM of 610 nm; (**f**) Focal spot of the micro-cylinder with a FWHM of 420 nm; (**g**) Focal spot of the hexagonal prism with a FWHM of 380 nm; (**h**) Focal spot of the rectangular pyramid with a FWHM of 400 nm.

Focusing and imaging are two functions of imaging systems, and they are two aspects of the same problem. The smaller the focal spot means the higher imaging resolution of the microscopic system. As the simulation on nano-focusing is relatively simple, which could be calculated with the FDTD algorithm for any shape of microparticle, we will firstly simulate the nano-focusing characteristics of the microparticle lens to find the appropriate structural parameters, and then carry out the imaging simulation. In the previous text, we proposed that the super-narrow focusing and super-resolution imaging abilities of a microsphere lens are caused by their near-field diffraction, so that these abilities are not unique to the microsphere, but are the characteristics of all microparticles. Here, four shapes of microparticle lenses’ focusing characteristics are simulated when illuminated by an *x* polarized plane wave along the negative direction of *z*-axis with wavelength of 550 nm. As illustrated in Figure 3a–d, a micro-cube, a micro-cylinder, a hexagonal prism, and a rectangular pyramid each form a focal spot below the microparticle, respectively. In each subgraph, the *xy* plane with maximum intensity is defined as the focal plane (FP) of the microparticle lens, represented by a white dotted line in the *xz* plane. Although the focal lengths and the focal spot sizes are different, there must always be a focal spot under the microparticle lens, and the focal spot parameters are adjustable by their RI. Usually, a higher RI means a smaller focal spot size and a shorter focal length, which usually increase or decrease simultaneously. The focal spot even shrinks into the microparticle when its RI is larger than the threshold value. This is different with a conventional objective lens, which needs a long focal length and work distance. The microparticle lens is placed at the sample surface when used to assist microscope for nano-imaging, which requires its focal spot to be located at the lower surface of microparticle lens.

To compare the focusing ability of each microparticle lens, we investigated the two most important parameters of the focal spot (the FWHM and sidelobe intensity) through the light intensity distribution at the focal plane. The former determines the imaging resolution and the latter determines the image quality. As illustrated in Figure 3e–h, we plotted the light intensity along the white dotted line passing through the center of the focal spot to measure the FWHM and the relative intensity of the sidelobes, as represented by the white solid line in the lower part of each subgraph. From the simulation results, it can be concluded that the FWHM of the focal spot and the relative intensity of the sidelobes depend on the size, shape, and refractive index distribution of the microparticle lens. Therefore, the optimization of the microparticle lens can be achieved by adjusting the above parameters to realize a smaller focal spot and a higher imaging resolution. In addition, the distribution of the sidelobes is caused by the near-field diffraction of the microparticle and determined by the shape of the microparticle. If the microparticle’s shape is rotationally symmetrical along the optical axis, its focal spot and sidelobes are also rotationally symmetrical, which means that it has the same imaging resolution in all directions. Otherwise, the focal spot and its sidelobes are not rotationally symmetrical, so the imaging resolution will be different in different directions. Therefore, in the following microparticle lens design, we will take a rotationally symmetrical microsphere as the basic structure, optimize its structural parameters to obtain super-narrow focusing and super-resolution image, and ensure that the imaging resolution is uniform in all directions.

**Figure 4:**
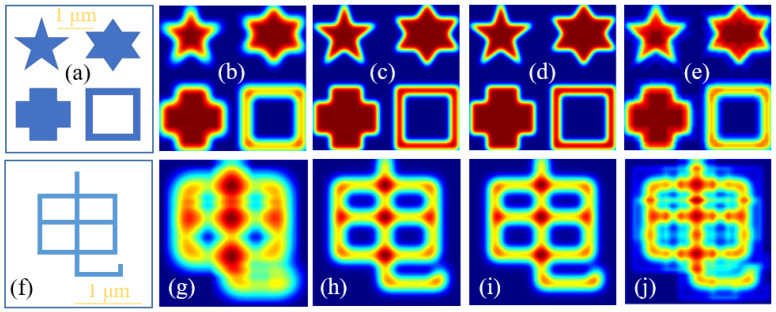
Nano-imaging of the above microparticle lenses. (**a**,**f**) are imaging targets; (**b**,**g**) are images obtained by the micro-cube; (**c**,**h**) are images obtained by the micro-cylinder; (**d**,**i**) are images obtained by the hexagonal prism; (**e**,**j**) are images obtained by the rectangular pyramid.

Although the focal spots of these microparticle lenses are not perfect, they can still be used for imaging. Here, we will use two samples to demonstrate their imaging effects. The samples are shown in the Figure 4a,f, which are some shapes and a Chinese character. With the micro-cube, the lines in this character are indistinguishable and the shapes’ profiles are blurred, illustrated in Figure 4b,g, as its focal spot is bigger than the feature size of imaging target. When imaged by the micro-cylinder and hexagonal prism, clearer images are obtained with smaller focal spots, as shown in Figure 4c,d,h,i. If imaged by the rectangular pyramid, the imaging results become very blurred due to the existence of ghosting, illustrated in Figure 4e,j, due to the high intensity of sidelobes. Therefore, it can be found that the larger focal spot, the lower the resolution. Meanwhile, the increase in sidelobe intensity can lead to the appearance of ghosting and image quality degradation.

## 4. Design and Optimization of Microparticle Lens

**Figure 5:**
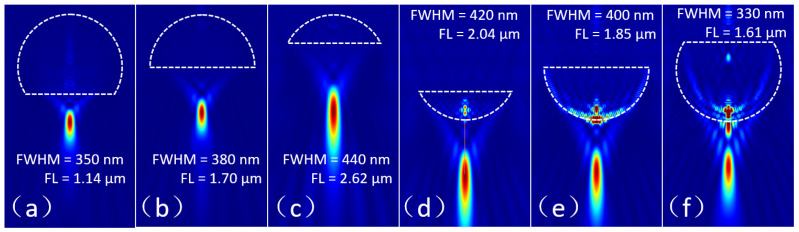
Influence of the height of the microsphere segment on its focal length and focal spot size. (**a**–**c**) Upper segments of the microsphere with heights of 3 μm, 2 μm, and 1 μm, respectively; (**d**–**f**) lower segments of the microsphere with heights of 1 μm, 2 μm, and 3 μm, respectively.

In this section, three variant structures of microsphere are proposed as microparticle lenses and their focusing characteristics are analyzed, especially the parameters of focal length and the FWHM. The first structure we investigated is the microsphere segment. Here, a microsphere (D = 4 μm, n = 1.5) is cut into segments with different heights as a microparticle lens. When the upper microsphere segments with heights of 3 μm, 2 μm, and 1 μm are illuminated by a plane wave with a wavelength of 400 nm, their focal spots are illustrated in Figure 5a–c, respectively. Obviously, as the microsphere segment height decreases, the focusing ability worsens gradually, the focal spot becomes larger gradually, and the focal length becomes longer synchronously. The same rule applies to the lower microsphere segment, as shown in Figure 5d–f. Therefore, by modulating the height and refractive index of the microsphere segment, the focal spot can be located exactly on its lower surface. Meanwhile the minimum focal spot can be achieved.

**Figure 6:**
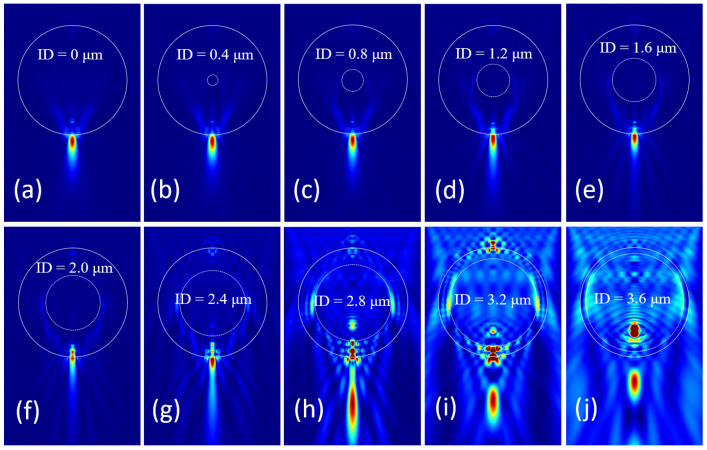
Simulation on the influence of the hollow microsphere’s inner diameter. (**a**) A solid microsphere with diameter of 4 μm and RI of 1.5; (**b**–**j**) hollow microspheres with ID of 0.4 μm, 0.8 μm, 1.2 μm, 1.6 μm, 2.0 μm, 2.4 μm, 2.8 μm, 3.2 μm, and 3.6 μm, respectively.

Another variant structure is a hollow microsphere, whose inner part is a concentric sphere filled with air. The focusing of a series of hollow microspheres is demonstrated in Figure 6. When we increase the inner diameter (ID) of the hollow microsphere from 0 μm to 3.6 μm with 0.4 μm per step, the focal spot shrinks toward the lower surface of microsphere, and the FWHM reduces gradually until the critical point appears. When the whole focal spot moves into the microsphere, another much weaker focal spot gradually forms outside the microsphere, as illustrated in Figure 6h. If the ID of the hollow microsphere continues to increase, the focal spot will gradually approach the lower surface of the microsphere again, as shown in Figure 6h–j. Through the modulation of this parameter, the smallest focal spot can be obtained with appropriate ID when the focal spot just locates at the lower surface of hollow microspheres.

**Figure 7:**
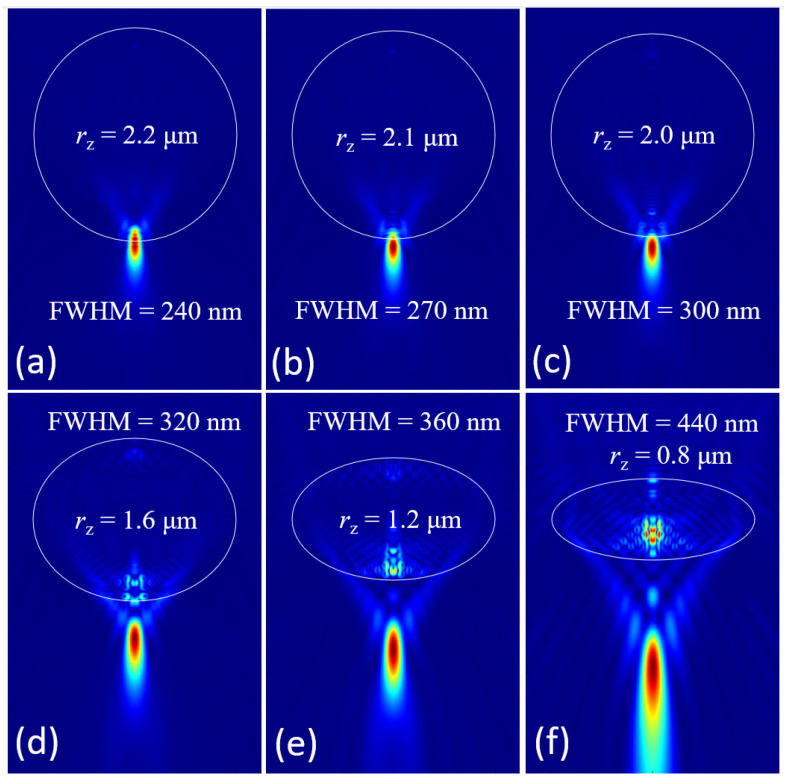
Focusing characteristics of the ellipsoid microparticle lens with different *r_z_*. (**a**,**b**) prolate ellipsoids with *r_z_* of 2.2 μm and 2.1 μm, respectively; (**c**) perfect microsphere lens with *r_x_* = *r_y_* = *r_z_* = 2 μm; (**d**–**f**) oblate ellipsoid with *r_z_* of 1.6 μm, 1.2 μm, and 0.8 μm, respectively.

The last structure we investigated is an ellipsoid with different radii along the optical axis direction, represented by *r_z_*. A standard microsphere with a radius of 2 μm and an RI of 1.5 can focus the incident plane wave into a focal spot outside the microsphere, as illustrated in Figure 7c. With the decrease of the *r_z_*, the focusing performance of the ellipsoid lens is weakening, its focal spot is getting larger gradually, and its focal length is also getting longer synchronously, illustrated in Figure 7d–f. On the contrary, when the *r_z_* is increased, the focal spot becomes smaller and shrinks toward the microparticle lens’ lower surface, as demonstrated in Figure 7a,b. When *r_z_* = 2.2 μm, the focal spot center is just located at the lower surface of ellipsoid; meanwhile, the focal spot is the smallest one which could be applied for microparticle lens assisted nanoscopy for a better resolution. Therefore, the oblateness of ellipsoid is another parameter that can adjust the size and position of the focal spot.

## 5. Comparison on the Imaging Properties of Three Types of Microparticle Lenses

**Figure 8:**
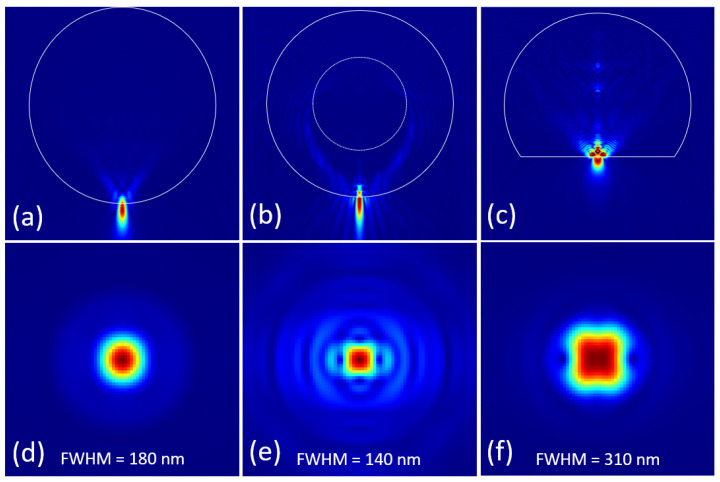
Nano-focusing of three types of microparticle lenses. (**a**) ellipsoid lens with an RI of 2.2 immersed in a liquid with an RI of 1.5; (**b**) hollow microsphere and its focal spot; (**c**) microsphere segment and its focal spot; (**d**–**f**) detailed presentation of the focal spots generated by these three kinds of microparticle lenses.

According to the conclusions summarized above, we proposed three types of microparticle lenses by adjusting and optimizing the above parameters respectively. The first one is an ellipsoid microparticle lens (*r_x_* = *r_y_* = 2.0 μm and *r_z_* = 2.2 μm) with an RI of 2.2 immersed in a surrounding medium with an RI of 1.5, as illustrated in Figure 8a, whose focal spot is just located at the lower surface of ellipsoid. If an area of 1 μm × 1 μm is selected in the center of the focal plane, the feature size and sidelobes of the focal spot can be displayed in detail, such as Figure 8d, whose FWHM is around 180 nm. Another microparticle lens is a hollow microsphere (out diameter is set to 4 μm) with an RI of 2.2 immersed in a liquid with an RI of 1.5, whose focal spot is illustrated in Figure 8b. When the ID is adjusted to 2 μm, its focal spot just moves to the surface of hollow microsphere, and the smallest focal spot with the FWHM of 140 nm appears, as shown in Figure 8e. The last structure is a microsphere segment with an RI of 2.2 immersed in air, as shown in Figure 8c, whose focal spot is also adjusted to the lower surface by adjusting the segment heigh to 3.0 μm. Its focal spot has been demonstrated in detail, as illustrated in Figure 8f, whose FWHM is around 310 nm. Compared with the incident wavelength of 400 nm, these three kinds of microparticle lenses all achieved sub-wavelength focusing, in which the focal spot of the hollow microsphere is the smallest and the sidelobes of the ellipsoid microparticle lens is the weakest.

**Figure 9:**
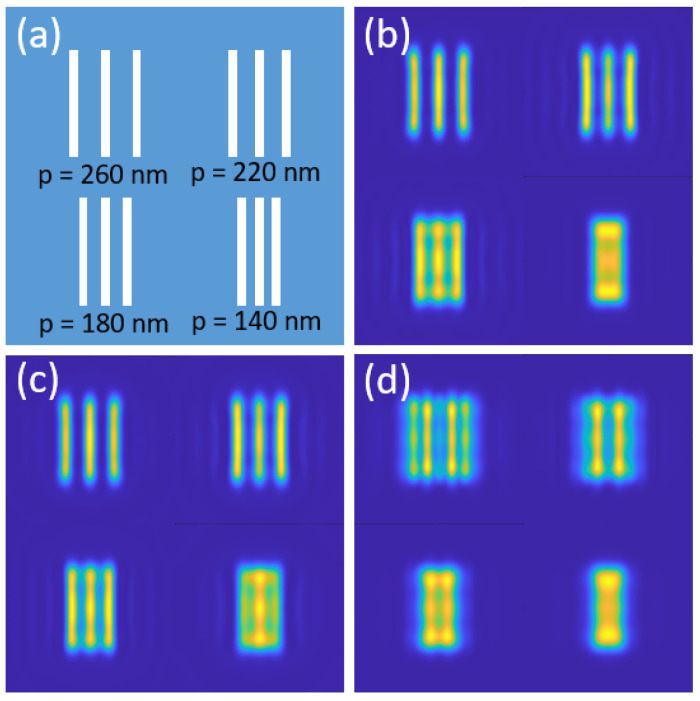
Super-resolution imaging ability of microparticle lenses. (**a**) Imaging target of three-bar structure with different periods; (**b**) imaging results of ellipsoid microparticle lens; (**c**) images captured by hollow microsphere; (**d**) imaging results of microsphere segment.

When applied to assisting the imaging of microscopical objective lens (NA = 0.9), these three kinds of microparticle lenses show great super-resolution imaging ability. In order to quantitatively analyze the imaging resolution, a series of three-bar structures with different periods are used as imaging targets, as shown in Figure 9a. When observed by the above designed ellipsoid microparticle lens, three periods of three-bar structures are imaged clear, while the smallest period of the structure (p = 140 nm) is indistinguishable, which means its minimum resolvable distance is around 180 nm. While the hollow microsphere lens is applied for imaging with the same immersion conditions, it could assist the objective lens to improve imaging resolution significantly, and even the minimum size of three-bar structure with period of 140 nm is resolved barely, as shown in Figure 9c. The last microparticle lens employed here is a microsphere (D = 4 μm) segment with an RI of 1.5 and a height of 3 μm whose imaging results are illustrated in Figure 9d, where all the four periods of the three-bar structures are indistinguishable as its FHWM and the minimum resolvable distance is around 310 nm, which is a little larger than the biggest periods of these three-bar structures.

The above simulations verified the super-resolution imaging characteristics of microparticle lenses and show the feasibility of the continuous optimization and upgrading of microparticle lenses for a smaller focal spot and better resolution. With the hollow microsphere lens, we have achieved a super-narrow focal spot with the FWHM of 140 nm and resolved the three-bar structure with a 140 nm period, which is equivalent to a resolution of 70 nm. In the following study, we will continue to design new structures of microparticle lenses based on the existing optical materials and continuously improve the imaging resolution and imaging quality.

## 6. Conclusions

A complete imaging simulation model for microparticle assisted nanoscopy was built for the design and optimization of microparticle lenses. Firstly, we demonstrated the nano-focusing characteristics with several non-spherical microparticles and revealed that the sub-wavelength focusing and super-resolution imaging characteristics are not exclusive to microspheres, which means that microsphere may not necessarily be the best choice for the highest imaging resolution. By adjusting the structural parameters, we found a method to compress the focal spot, which can be used to improve the imaging performance of microparticle lenses. Based on the above experiences, we designed three types of microparticle lenses and demonstrated their nano-focusing and nano-imaging ability numerically. The modified microparticle lenses have smaller focal spots and a higher imaging resolution than those of the microsphere lens, which obtained a smallest FWHM of 140 nm at focal plane and a highest resolution of around 70 nm (~λ/6). Due to the advantages of being label free, being compatible with conventional optical microscope, and neither imaging post processing nor laser illuminating being needed, this approach is easy to implement and valid for diverse samples. Therefore, it can find use in a variety of applications, such as label-free biological imaging and surface defect detection as well as other fields requiring real-time nanoscale visualization.

## Data Availability

Data are available on request from the authors.
